# Irreversibility of cellular senescence: dual roles of p16^INK4a^/Rb-pathway in cell cycle control

**DOI:** 10.1186/1747-1028-2-10

**Published:** 2007-03-07

**Authors:** Akiko Takahashi, Naoko Ohtani, Eiji Hara

**Affiliations:** 1 Division of Protein Information, Institute for Genome Research, University of Tokushima, 3-18-15 Kuramoto-cho, Tokushima 770-8503 Japan

## Abstract

The retinoblastoma (Rb) tumor suppressor gene product, pRb, has an established role in the implementation of cellular senescence, the state of irreversible G1 cell cycle arrest provoked by diverse oncogenic stresses. In murine cells, senescence cell cycle arrest can be reversed by subsequent inactivation of pRb, indicating that pRb is required not only for the onset of cellular senescence, but also for the maintenance of senescence program in murine cells. However, in human cells, once pRb is fully activated by p16^INK4a^, senescence cell cycle arrest becomes irreversible and is no longer revoked by subsequent inactivation of pRb, suggesting that p16^INK4a^/Rb-pathway activates an alternative mechanism to irreversibly block the cell cycle in human senescent cells. Here, we discuss the molecular mechanism underlying the irreversibility of senescence cell cycle arrest and its potential towards tumor suppression.

## Background

Cellular senescence is the state of stable cell cycle arrest provoked by a variety of potentially oncogenic stimuli, such as telomere shortening, DNA damage or activation of certain oncogenes [1-3]. Cellular senescence appears to be acting as a barrier to cancer, preventing damaged cells from undergoing aberrant proliferation [4-10]. Two well established tumor suppressor proteins, pRb and p53, have been shown to play key roles in cellular senescence [1-3]. The activities of pRb and p53 are dramatically increased during cellular senescence and inactivation of these proteins in senescent mouse embryonic fibroblasts (MEFs) results in the reversal of the senescence phenotype leading to cell cycle re-entry, suggesting that pRb and p53 are required not only for the initiation of senescence program but also for the maintenance of the senescence state in murine cells [1-3,11,12]. In human senescent cells, however, once pRb is fully engaged, particularly by its activator p16^INK4a^, senescence cell cycle arrest become irreversible and is no longer revoked by subsequent inactivation of pRb and p53 [13-15]. Interestingly, subsequent inactivation of pRb and p53 enables human senescent cells to reinitiate DNA synthesis but fails to drive the complete cell cycle, suggesting that these cells may be arrested in G2 or M phase of the cell cycle [13,14]. This pRb- and p53- independent cell cycle block, which seems to be specific for human cells, is likely to act as a second barrier to cellular immortalization and may help to explain the remarkable stability of the senescence cell cycle arrest in human cells [2,15]. Recent work in our lab has uncovered an unexpected role for the p16^INK4a^/Rb-pathway and provided a new insight into how senescent cell cycle arrest is enforced in human cells [16]. In this commentary, we will take a closer look at the genes and mechanism involved.

## The G1/S control in cellular senescence

In higher eukaryotes, pRb is a crucial gatekeeper of cell cycle progression [17-21]. The activity of pRb is tightly regulated by various post-translational modifications, such as phosphorylation, acetylation and ubiquitination, and is thought to impose a block on G1 progression that is alleviated by phosphorylation [17-21]. In particular, a series of cyclin-dependent kinases (CDKs), CDK2, CDK4 and CDK6, play a critical role in the phosphorylation of pRb [18,22-25]. When pRb is phosphorylated by these CDKs, pRb loses its ability to bind E2F/DP transcription factor complexes resulting in entry into S-phase of the cell cycle [26-28]. However in senescent cells, the activity of CDKs is blocked by elevated expression of CDK inhibitors, p21^Cip1/Waf1/Sdi1 ^and p16^INK4a ^[29-32]

p21^Cip1/Waf1/Sdi1 ^is a founding member of the mammalian CDK inhibitor family and is one of the best characterized transcriptional targets of the p53 tumor suppressor protein [29,33-36]. Thus, p21^Waf1/Cip1 ^links the p53- pathway to the Rb- pathway, providing a tight security network towards tumor suppression. Indeed, the role of p21^Cip1/Waf1/Sdi1 ^expression is well documented in various cell culture studies; up-regulation of p21^Cip1/Waf1/Sdi1 ^expression participates in processes such as DNA damage-induced cell cycle arrest, cellular senescence and terminal differentiation that may prevent tumor formation [22]. However, since mutations in the *p21*^*Waf1*/*Cip1*/*Sdi1 *^gene are rarely observed in human cancers and mice lacking *p21*^*Waf1*/*Cip1*/*Sdi1 *^gene do not exhibit any predisposition to spontaneous tumor formation [37-40], it remains unclear whether p21^Cip1/Waf1/Sdi1 ^indeed plays a key role in tumor suppression *in vivo*.

The *INK4a *gene encodes another type of CDK inhibitor, p16^INK4a^, which specifically binds to and inactivates D-type CDKs, CDK4 and CDK6 [41]. The binding of p16^INK4a ^to CDK4/6 also induces redistribution of Cip/Kip family CDK inhibitors, p21^Cip1/Waf1/Sdi1 ^and p27^Kip1^, from cyclinD-CDK4/6 to cyclinE-CDK2 complexes resulting in the inactivation of CDK2-kinase [22,42,43]. Thus, induction of p16^INK4a ^collaborates with p21^Cip1/Waf1/Sdi1 ^to prevent phosphorylation of pRb, leading to a stable G1 arrest in senescent cells [32]. Importantly, the p16^INK4a ^gene is frequently inactivated in a wide range of human cancers and is therefore recognized as a tumor suppressor gene [32]. This may also be because the coding region of the p16^INK4a ^gene is partly shared with another tumor suppressor gene called p14^ARF ^(also called as p19^ARF ^in mouse) [32,44,45]. In human cancer, however, a large number of the point mutations within this region only affect p16^INK4a ^activity but not p14^ARF ^activity, indicating that p16^INK4a ^/Rb-pathway, in itself, also play key roles in tumor suppression [32].

## Cytokinetic block: a second barrier in cellular senescence

Although p16^INK4a ^is known to exert its effects through pRb, subsequent inactivation of pRb stimulates DNA synthesis but not cell proliferation if p16^INK4a ^is ectopically expressed prior to inactivation of pRb in human cells [14]. By contrast, inactivation of pRb is sufficient to override the p16^INK4a ^effect if pRb is inactivated prior to p16^INK4a ^expression [14]. It is therefore likely that once pRb is fully activated by p16^INK4a^, pRb activates yet another mechanism that irreversibly causes cell cycle arrest either in G2 or M phase [2,13,14]. Indeed, a dramatic increase of poly-nucleated cells is observed when pRb and p53 were subsequently inactivated in human cells expressing high level of p16^INK4a ^[16], suggesting that this mechanism may target cytokinesis.

To delineate the molecular events underlying this cytokinetic block in human senescent cells, we took advantages of using SVts8 cells, a conditionally immortalized human fibroblasts cell lines that express a temperature-sensitive (*ts*) mutant of simian virus 40 large T antigen (LT) and elevated level of endogenous telomerase [46,47]. Using SVts8 cells, we were able to examine the irreversibility of senescence cell cycle arrest under various different conditions and have shown that p16^INK4a^/Rb-pathway cooperate with mitogenic signals to enforce irreversible cytokinetic block through activating production of reactive oxygen species (ROS) [16].

**Figure 1:**
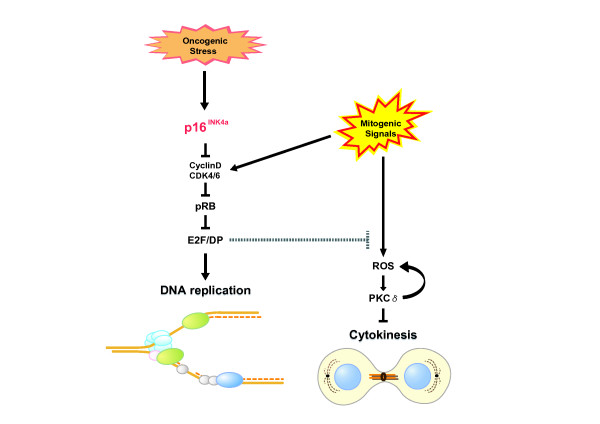
**The roles of p16**^INK4a^**/RB-pathway in senescence cell cycle arrest. **In proliferating cells, the effects of mitogenic signals in ROS production are counterbalanced by E2F/DP activity. However, when E2F/DP activity is shut down by fully activated pRb, mitogenic signaling, in turn, increases the level of ROS and elicits a positive feedback activation of ROS/PKC-δ signaling pathway. Elevated levels of p16^INK4a ^therefore establish an autonomous activation of ROS/PKC-δ signaling, leading to an irrevocable block to cytokinesis in human senescent cells.

Although ROS are required for the physiological function of the cells, excessive ROS cause anti-proliferative effects such as apoptosis and/or cellular senescence [48]. During low stress condition, mitogenic signals inactivate pRb and therefore activate E2F/DP complexes to stimulate S-phase entry [22,26-28]. Moreover, E2F/DP activation decrease ROS levels by regulating genes involved in ROS production [16]. Thus, although mitogenic signals have the potential to stimulate ROS production, this effect appears to be counterbalanced by E2F/DP activity in proliferating normal human cells [16]. In condition of high cellular stress, however, the activity of E2F/DP is blocked by p16^INK4a^/Rb-pathway. In this setting, mitogenic signaling, in turn, increases the ROS production, thereby activating PKCδ, a critical downstream mediator of the ROS signaling pathway [16,49,50]. Importantly moreover, once, activated by ROS, PKCδ, promotes further generation of ROS, thus establishing a positive feed back loop to sustain ROS- PKCδ signaling [16]. Sustained activation of ROS- PKCδ signaling irreversibly blocks cytokinesis, at least partly through reducing the level of WARTS (also known as LATS1), a mitotic exit network (MEN) kinase required for cytokinesis [51-53], in human senescent cells [16]. Thus, elevated levels of p16^INK4a ^establish an autonomous activation of ROS- PKCδ signaling, leading to an irrevocable block to cytokinesis in human senescent cells (see model in Figure 1). This system may serve as a fail-safe mechanism, especially in case of the accidental inactivation of pRb and p53 in human senescent cells [15,16]. It is noteworthy that we were unable to see activation of PKCδ during replicative senescence in MEFs [16]. This difference may account for the reversibility of murine cell senescence.

## Concluding remarks

Although we can not rule out the possibility that other mechanisms might also involved in the irreversible senescence cell cycle arrest [54-59], our results reveal a novel activity of the p16^INK4a^/Rb- pathway and facilitate our understanding of how cellular senescence is securely controlled in human primary cells. Understanding the strict irreversibility of cellular senescence will provide valuable new insights into the development of cancer and open up new possibilities of its control [60-62].

## Abbreviations

CDKs: cyclin dependent kinases

pRb: the retinoblastoma tumor suppressor gene product

*ts*: temperature sensitive

LT: simian virus 40 large T antigen

MEFs: mouse embryonic fibroblasts

ROS: reactive oxygen species

MEN: mitotic exit network

## Competing interests

The author(s) declare that they have no competing interests.
